# Glutaraldehyde Cross-linking of HIV-1 Env Trimers Skews the Antibody Subclass Response in Mice

**DOI:** 10.3389/fimmu.2017.01654

**Published:** 2017-11-27

**Authors:** Martina Soldemo, Monika Àdori, Julian M. Stark, Yu Feng, Karen Tran, Richard Wilson, Lifei Yang, Javier Guenaga, Richard T. Wyatt, Gunilla B. Karlsson Hedestam

**Affiliations:** ^1^Department of Microbiology, Tumor and Cell Biology, Karolinska Institutet, Stockholm, Sweden; ^2^Department of Immunology and Microbial Science, Neutralizing Antibody Center, International AIDS Vaccine Initiative, The Scripps Research Institute, La Jolla, CA, United States

**Keywords:** HIV-1 env, gluteraldehyde, cross-linking, immunogenicity, mice, vaccine responses, antibody isotypes

## Abstract

Well-ordered soluble HIV-1 envelope glycoprotein (Env) spike mimetics such as Native Flexibly Linked (NFL) trimers display high homogeneity, desired antigenicity, and high *in vitro* stability compared to previous generation soluble HIV-1 Env trimers. Glutaraldehyde (GLA) cross-linking was shown to further increase the thermostability of clade C 16055 NFL trimers and enhance the induction of tier 2 autologous neutralizing antibodies in guinea pigs. Here, we investigated if GLA fixation affected other aspects of the Env-specific immune response by performing a comparative immunogenicity study in C57BL/6 mice with non-fixed and GLA-fixed 16055 NFL trimers administered in AbISCO-100 adjuvant. We detected lower Env-specific binding antibody titers and increased skewing toward Th2 responses in mice immunized with GLA-fixed trimers compared to mice immunized with unfixed trimers, as shown by a higher Env-specific IgG1:IgG2b antibody subclass ratio. These results suggest that the presence of GLA adducts on Env influences the quality of the induced antibody response.

## Introduction

Most licensed vaccines mediate protection through the induction of highly specific IgG serum antibodies. Consequently, a central goal for HIV-1 vaccine development is to induce antibody responses that are capable of neutralizing a broad range of circulating HIV-1 strains. Over the past decades, HIV-1 envelope glycoprotein (Env) immunogen design efforts have focused on the generation of recombinant, soluble trimeric Env variants consisting of the exterior glycoprotein, gp120, and the ectodomain of the transmembrane protein, gp41, such as the foldon trimers and the SOS trimers (1, 2). As is now appreciated, these early generation trimers were structurally heterogeneous and suboptimal antigenic mimics of the functional HIV-1 spike.

More recently, new generation trimers such as the BG505 SOSIP.664 trimers (3, 4) and various forms of the native flexibly linked (NFL) trimers (5) were designed. These soluble spikes display superior threefold symmetric order and improved antigenic profiles. The SOSIP trimers were the progenitors, containing an internal cysteine linkage between gp120 and gp41 and an isoleucine (I) to proline (P) change in gp41 (I559P) to disfavor the post-fusion conformation of HIV-1 Env (2). The NFL trimers were constructed by replacing the furin cleavage site that is naturally present between the exterior glycoprotein gp120 and the transmembrane protein gp41 with a flexible linker composed of two repeats of four glycine and one serine residues (G_4_S)_2_. This modification renders these trimers cleavage- and furin-independent, forming a covalent linkage between gp120 and the ectodomain of gp41 (5–7). Besides the I559P change, further developments of the NFL trimers included the introduction of a set of substitutions selected from the BG505 Env sequence, referred to as trimer-derived (TD), which favor the production of trimers that display ordered symmetry and desired antigenic profiles and can be applied to diverse HIV-1 strains (6). Additional modifications of the NFL TD trimer design performed in the context of the Indian clade C isolate 16055 was the introduction of a cysteine bond between residues I201C and A433C to retain gp120 in the pre-CD4-bound conformation, resulting in the 16055 NFL TD CC trimers (6). A set of glycine substitutions in selected gp41 coil-to-helix transition residues were also introduced to further stabilize the pre-fusion state (8).

In addition to efforts using targeted mutagenesis of Env to improve trimer stability, glutaraldehyde (GLA) cross-linking was shown to improve the thermostability of HIV-1 Env trimers as well as the induction of neutralizing antibody responses (7, 9, 10). Depending on the specific Env construct used, negative or positive selection of the trimers may be required prior to fixation to enrich for conformers with desired antigenicity. While intramolecular protein cross-linking may provide a benefit in terms of increasing the durability of conformationally sensitive neutralizing antibody epitopes *in vivo*, less is known about whether fixation affects other aspects of the Env-specific immune response. In this study, we addressed this issue by immunizing C57BL/6 mice with either fixed or unfixed 16055 NFL TD CC trimers formulated in AbISCO-100 adjuvant. After the first boost, we observed an overall reduction in Env-specific serum-binding antibody titers in mice inoculated with fixed trimers compared to mice inoculated with unfixed trimers, which was primarily detected when coating was performed with unfixed trimers. However, this difference was modest when fixed trimers were used for coating and especially after an additional boost. We further detected a pronounced skewing toward Th2 responses with significantly altered Env-specific IgG1:IgG2b ratios in the sera of mice immunized with GLA-fixed trimers compared to mice immunized with unfixed trimers. A similar effect was detected for the IgG1:IgG2c ratios and a trend toward increased production of Th2 cytokines from stimulated CD4+ T cells was observed in mice immunized with fixed trimers. These results demonstrate that protein cross-linking influences the induced antibody responses at several levels *in vivo*.

## Materials and Methods

### Animals, Immunizations and Reagents

Male C57BL/6 Bom mice were purchased from Taconic, Denmark. Mice were immunized subcutaneously with 10 µg of recombinant 16055 NFL TD CC trimers together with 10 µg AbISCO-100 adjuvant (Isconova/Novavax) or with adjuvant alone. The mice were 7–9 weeks of age at the start of the immunizations, and booster immunizations were performed at 4-week intervals. All mice were kept at the animal facility of the Department of Microbiology, Tumor and Cell Biology at Karolinska Institutet. All animal experiments were performed under approved conditions and standard guidelines prior to the experimental start according to the regulations of the Committee for Animal Ethics (Stockholm, Sweden).

### Expression and Purification of Soluble Env Trimers

The 16055 trimers were produced as previously described (5, 6). Briefly, the trimers were expressed in 293F cells and were isolated by lectin-affinity chromatography using GNL (*Galanthus nivalis* lectin-agarose; Vector Labs), purified by size-exclusion chromatography (SEC) using Superdex™ 200 columns (GE Healthcare Life Sciences) to isolate the predominant trimeric fractions and further purified by negative selection affinity chromatography using the non-neutralizing CD4bs-directed mAb, GE136 (11).

### Trimer Cross-linking and Gel Analysis

Cross-linking of the purified 16055 trimers was conducted as previously described (7). Briefly, 0.5 mg/ml of trimer was fixed with 5 mM GLA (ACROS Organics) at room temperature (RT) for 5 min and then the reaction was quenched by excess 50 mM glycine, pH 7.5. The fixed trimers were negatively selected by GE136 antibody affinity chromatography and re-isolated by Superdex™ 200 size-exclusion chromatography and then analyzed by SDS-PAGE under reducing and non-reducing conditions and by Blue Native PAGE as described previously (7).

### Differential Scanning Calorimetry (DSC) and Negative-Stain Electron Microscopy (EM)

The thermal melting (Tm) of the trimers was determined using a Microcal VP-Capillary DSC (Malvern). Briefly, trimers were diluted in PBS pH 7.4 to 0.25 mg/ml and scanned at a rate of 1°C/min. Data collected were analyzed after buffer correction, normalization, and baseline subtraction using the VP-Capillary DSC Automated data analysis software. For EM analysis, the 16055 NFL unfixed and fixed trimers were negatively stained on glow-discharged carbon-coated copper mesh grids (Electron Microscopy Sciences) for 2 min. Following blotting to remove excess sample, grids were transferred onto droplets of 2% phosphotungstic acid (pH 6.7) for 2 min. Following blotting and drying the grids were analyzed on a Philips CM100 electron microscope and imaged at selected magnifications with a Megaview III charge-coupled-device camera.

### ELISA for Antigenic Profiling and Detection of Serological Antibody Responses

To assess binding by selected bNAbs and non-neutralizing mAbs, the 16055 NFL CC TD trimers were captured by their His-tag using a mouse anti-His antibody coated on the ELISA plate overnight (ON), followed by washing, blocking, and detection using anti-mouse IgG as described below. To detect Env-specific antibody responses in serum, 96-well high-protein-binding MaxiSorp (Nunc) plates were pre-coated with 1 µg/ml *Galanthus nivalis* lectin (Sigma) diluted in PBS and incubated ON at 4°C. Plates were then washed six times in washing buffer (PBS/0.05% Tween-20) followed by addition of 150 μl/well blocking buffer (2% fat-free milk in PBS) and incubated for 1 h at RT. After incubation, the blocking buffer was removed from the plates and 200 ng/well unfixed or fixed NFL Env trimers were added and let to incubate at RT for 2 h. Plates were washed six times in washing buffer and were then incubated in blocking buffer for 1 h. After removing the blocking buffer, sera were added to the plates in threefold serial dilution starting at 1:25 dilution in blocking buffer and incubated for 2 h at RT. After washing the plates six times in washing buffer, secondary antibody diluted in PBS was added to each well. For total, Env-specific IgG ELISA, the secondary antibody goat anti-mouse IgG-horse radish peroxidase (HRP) (Southern Biotech) was used in a dilution of 1:1,000. For subclass-specific Env serum antibody detection, goat anti-mouse IgG1-HRP (Southern Biotech) (1:5,000), goat anti-mouse IgG2b-HRP (Southern Biotech) (1:5,000), goat anti-mouse IgG2c-HRP (Southern Biotech) (1:5,000), or goat anti-mouse IgG3-HRP (Southern Biotech) (1:1,500) were added. Secondary antibodies were incubated at RT for 1 h and removed by washing six times in wash buffer. To develop plates, 100 μl/well of TMB stabilized chromogen substrate (Invitrogen) was added and incubated for 10 min in dark at RT. The reaction was stopped by adding 1 M H_2_SO_4_. The optical density was measured at 450 nm using an Asys Expert 96 ELISA reader (Biochrom).

### Preparation of Single Cell Suspension

The mice were sacrificed by cervical dislocation and spleens were taken out for further analysis. Single cell suspension of splenocytes was obtained by passing the dissociated spleen through a 70-µM nylon cell strainer. Hypotonic ammonium chloride solution was used to lyse the red blood cells. Splenocytes were then collected in complete RPMI 1640 medium (containing 5% FBS, 50 µM 2-mercaptoethanol, 2 mM l-glutamine, 100 U/ml penicillin, and 100 µM streptomycin), and cell numbers were calculated using the automated cell counter Countess (Invitrogen) for further experiments.

### CD4+ T Cell Depletion

To deplete CD4+ T cells, the protocol from EasySep negative selection kit was followed (Stemcell Technologies). Briefly, splenocytes were incubated with normal rat serum (Stemcell Technologies) and biotinylated rat anti-mouse CD4 antibody (clone: RM4-5; BD Pharmingen) for 10 min with mixing every third minute. EasySep Strepativin Rapid Sphere 50001 beads (Stemcell Technologies) were added to the cell mixture at a concentration of 75 µl/ml of cell suspension. CD4+ T cells were separated using an EasySep magnet (Stemcell Technologies) and the negative fraction was collected in a new tube and used for further experiments.

### Flow Cytometry

Total splenocytes and CD4-depleted cell fractions were stained on ice for 20 min with the following antibodies: CD3e-PE (145-2C11; eBioscience), CD8a-APC (53-6.7; BD Pharmingen), CD4-FITC (H129.19; BD Pharmingen), and B220-PerCP-Cy5.5 (RA3-6B2; BD Pharmingen). The samples were run on a FACSCalibur cytometer (BD Bioscience), and data were analyzed with FlowJo software version 10 (TreeStar).

### T Cell ELISpot Analysis

T cell ELISpot analysis was performed to measure cytokine production after stimulation of total splenocytes. 96-well Multiscreen-IP filter plates (Millipore) were pre-treated with 70% ethanol, washed three times in PBS followed by coating with 5 μg/well (50 µg/ml) of anti-mouse IFNγ (mAb: AN18), anti-mouse IL-2 (mAb: 1A12), or anti-mouse IL-4 (mAb: 11B11), all from Mabtech AB. Plates were incubated ON at 4°C. Before addition of splenocytes, the plates were washed six times with PBS/0.05% Tween-20 and blocked in complete RPMI medium for 2 h at 37°C/5% CO_2_ in a humidified incubator. After incubation splenocytes, in triplicates, were added to the wells in three different concentrations (200,000, 100,000, or 50,000 cells) in a final volume of 150 µl and stimulated with one of the following stimuli: ConA (2 µg/ml) (Sigma), unfixed or fixed NFL trimers (6.67 µg/ml), or left unstimulated in medium only. After 20 h stimulation at 37°C/5% CO_2_ in a humidified incubator, the cells were removed from the wells and the plates were washed six times with PBS/0.05% Tween-20. Then the following biotinylated secondary antibodies in a concentration of 1 µg/ml (Mabtech AB) were added to the corresponding wells: anti-mouse IFNγ (mAb: R4-6A2), anti-mouse IL-2 (mAb: 5H4), or anti-mouse IL-4 (BV06-24G2). After incubation at RT for 2 h, the plates were washed six times in PBS only and streptavidin-ALP (Mabtech AB) in a 1:1,000 dilution was added to wells and incubated at RT for 45 min. After washing with water, plates were developed with 100 μl/well of BCIP/NBT plus substrate (Mabtech AB) for 10 min at RT. To stop the reaction, wells were emptied and washed extensively in water followed by air-drying. The spots were counted in an ImmunoSpot analyzer (CTL Immunospot).

### Flow Cytometric Bead Array (CBA) to Detect Cytokines after *In Vitro* Stimulation

Total splenocytes from mice immunized three times were stimulated *in vitro* for detection of cytokine production. One million splenocytes were used for each stimulation in 48-well plates in a total volume of 500 µl. Each mouse was stimulated with either ConA (2 µg/ml) (Sigma), unfixed or fixed NFL trimers (6.67 µg/ml) or left unstimulated in medium only. The plates were incubated for 20 h at 37°C/5% CO_2_ in a humidified incubator. Plates were spun down, and supernatants were collected. To measure the secreted cytokine from each mouse and stimuli, the BD CBA Mouse Enhanced Sensitivity Master Buffer Kit (BD Bioscience) was used. IL-5, IL-10, and IL-13 (BD Bioscience) were measured in all samples according to manufacturer’s instruction. Standards were prepared from Top Standard by threefold dilutions down to 1:729. Each sample was diluted in two different dilutions, 1:2 and 1:20. Diluted samples were mixed and incubated with Capture Beads for 2 h in dark at 4°C. The samples were then washed in FACS Flow for 5 min at 300*g*. Supernatant was flicked off before Mouse Detection Reagent was added and incubated for 2 h in dark at 4°C. After an additional washing step, the Enhanced Sensitivity Detection Reagent was added. After 1 h incubation in dark at 4°C, the samples were washed and ran on FACSVerse (BD Bioscience). Standard curves were generated for each cytokine. The samples were then calculated based on the median fluorescence values. If the value was lower than the standard curve, those samples were considered as 0. Samples higher than the detection limit (based on standard curve) was excluded.

### Statistical Analysis

GraphPad Prism software version 8 (San Diego, CA, USA) was used to analyze data by Student’s *t*-test. Significance was defined as *p ≤ 0.05, **p ≤ 0.01, and ***p ≤ 0.001.

## Results

### *In Vitro* Characterization of Unfixed and Fixed Env Trimers

**Figure 1:**
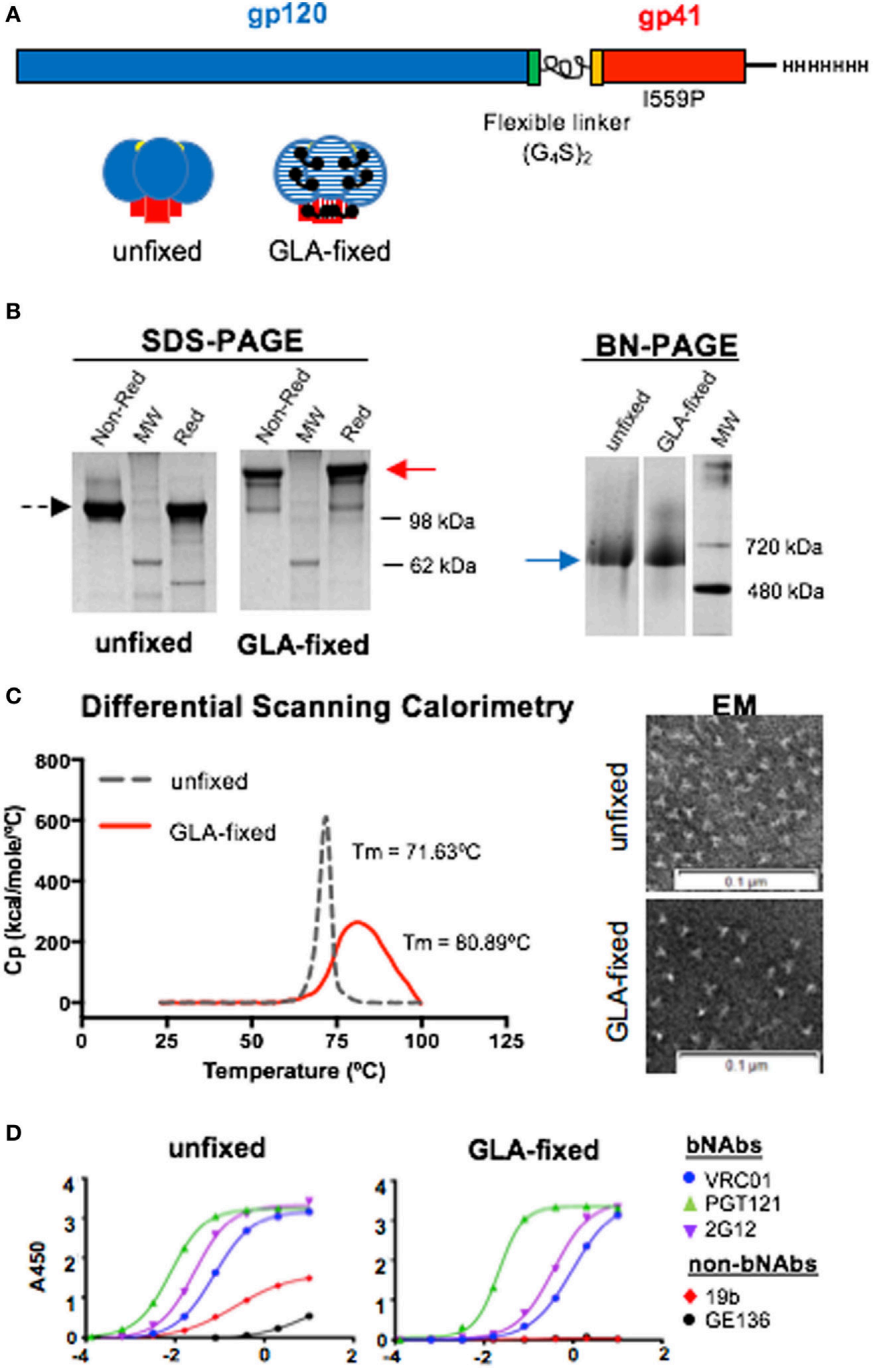
Schematic illustration and *in vitro* characterization of the 16055 native flexibly linked (NFL) trimer-derived (TD) CC Env trimers. **(A)** Linear representation of the NFL TD CC Env trimer sequence with the flexible (G_4_S)_2_ peptide linker indicated between gp120 and gp41 (top) and cartoon of the unfixed and glutaraldehyde (GLA)-fixed Env trimers (bottom). **(B)** Left panel: SDS gel of unfixed trimers under non-reducing conditions and reducing conditions with molecular weight (MW) marker shown in between; middle panel: SDS gel of GLA-fixed under non-reducing conditions and reducing conditions with MW marker shown in between; right panel: blue native gel of unfixed and GLA-fixed trimers. **(C)** Left panel: differential scanning calorimetry curves comparing the *in vitro* stability of unfixed (dashed line) and GLA-fixed (solid line) 16055 NFL TD CC trimers; right panel: negative-stain electron microscopy (EM) images of unfixed and GLA-fixed 16055 NFL TD CC trimers. **(D)** ELISA comparing the antigenic profile using a set of Env-specific monoclonal antibodies of the unfixed (left) and GLA-fixed (right) 16055 NFL TD CC trimers.

In this study, we used the well-ordered 16055 NFL TD CC Env trimers (Figure 1A) to investigate the effect of GLA fixation on Env-specific immune responses in C57BL/6 mice. Following the cross-linking procedure, the fixed trimers were isolated by negative selection and size-exclusion chromatography (SEC). Analysis of the purified GLA-fixed trimers by reducing SDS-PAGE confirmed that cross-linking of the trimers had occurred, by a shift in the apparent molecular weight (MW) relative to the unfixed trimers. GLA cross-linking rendered the trimers resistant to disulfide-directed reduction (Figure 1B, left). BN-PAGE analysis, under native conditions, revealed that both the unfixed and fixed trimers migrated with similar MWs (Figure 1B, right). Both sets of data indicated that the cross-linking had occurred between protomers within each trimer, but not across individual trimers. These results were consistent with what we had reported previously by these types of analyses (7). To measure the thermal stability of the unfixed and fixed 16055 NFL TD CC Env trimers, we used DSC. The GLA-fixed Env trimers displayed a higher thermostability compared to the unfixed counterpart. The thermal denaturation midpoint temperature (Tm) differed nearly 10°C between the two proteins (Figure 1C, left), indicating that GLA cross-linking contributes to the overall stability of the protein. The increased Tm was accompanied by a broadening of the thermal transition profile, indicating some molecular heterogeneity following the GLA cross-linking process. To confirm that the trimers remained as single particles following cross-linking and negative selection, we performed negative-stain EM and observed no marked difference in trimers at this level of resolution comparing unfixed to fixed populations (Figure 1C, right). To confirm trimer concentrations and that selected epitopes were minimally affected following fixation, we performed ELISA using the bNAbs VRC01, PGT121, and 2G12. We observed that PGT121 recognition was not greatly affected, indicating that the protein concentrations were accurate, whereas there was some decrease in VRC01 and 2G12 recognition following GLA fixation. We included the non-neutralizing antibodies 19b and GE136 that poorly recognized the unfixed trimers, as expected, whereas recognition of the GLA-fixed trimers by these mAbs was completely eliminated (Figure 1D).

### Env-Specific Binding Antibody Responses in Mice Immunized with Unfixed or GLA-Fixed Trimers

**Figure 2:**
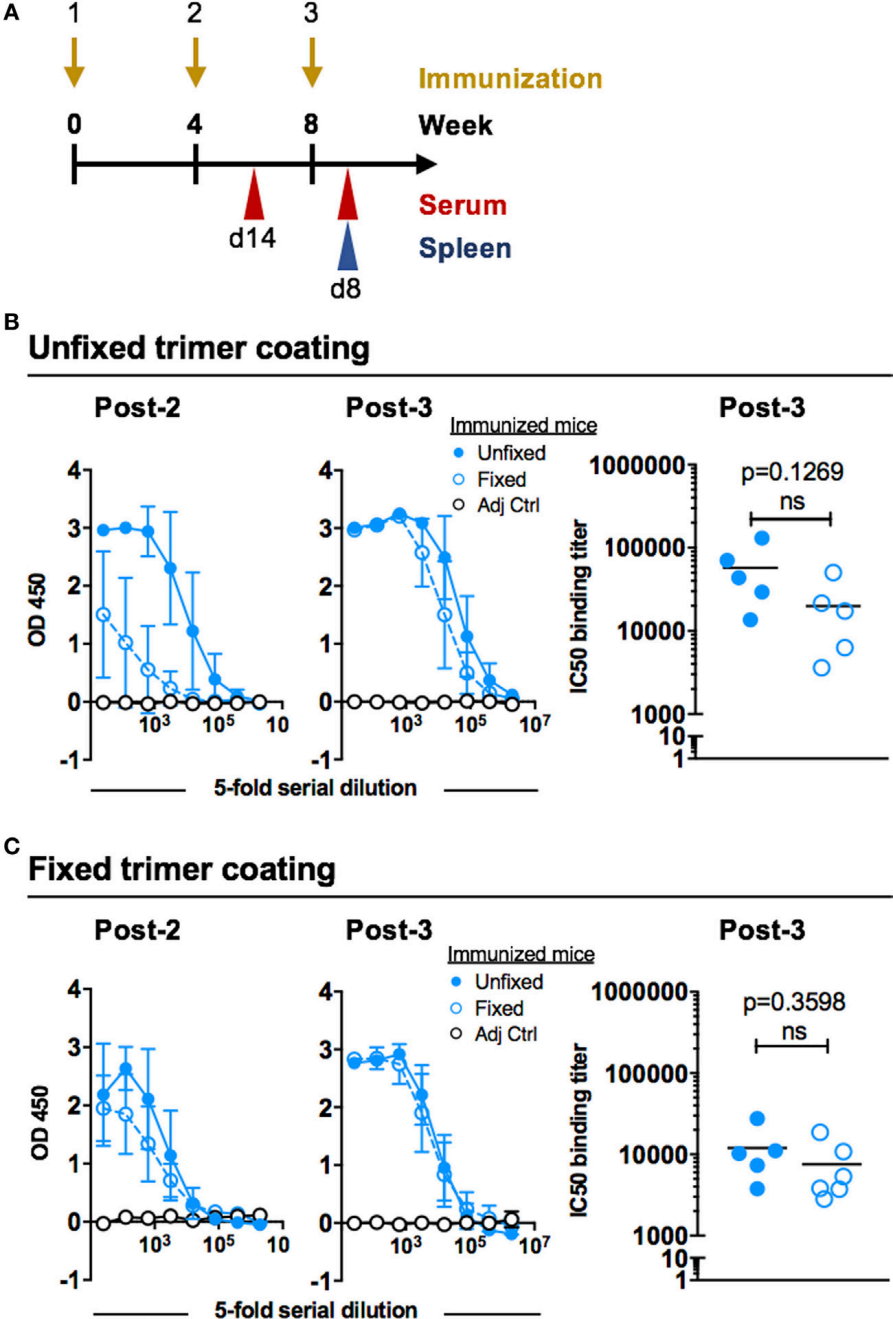
Immunization schedule and Env binding titers after inoculation with 16055 native flexibly linked (NFL) trimer-derived (TD) CC Env trimers. **(A)** C57BL/6 mice (six per group) were immunized at 0, 4, and 8 weeks with 10 µg of unfixed or glutaraldehyde-fixed 16055 NFL TD CC Env trimers together with 10 µg AbISCO-100 adjuvant, or with adjuvant alone (*n* = 2). Serum was collected 14 days following the second immunization and serum and spleens were collected 8 days following the third immunization. **(B)** Env-specific IgG binding titers were measured by ELISA after two or three immunizations using unfixed 16055 NFL TD CC Env trimers for coating: full titration curves (group means) are shown to the left and IC50 binding titers (individual animals) are shown for the post-3 serum to the right. **(C)** Env-specific IgG binding titers were measured by ELISA after two or three immunizations using fixed 16055 NFL TD CC Env trimers for coating: full titration curves are shown to the left (group means) and IC50 binding titers (individual animals) are shown for the post-3 serum to the right. Statistical significance (Student’s *t*-test) between post-3 IC50 titers in mice injected with unfixed or fixed trimers immunized mice was tested. Fivefold serial dilution was used for all samples starting at a 1:25 dilution.

To evaluate the immunogenicity of the unfixed and GLA-fixed 16055 NFL TD CC trimers C57BL/6 mice were immunized three times at 4-week intervals. Sampling was performed 2 weeks after the second immunization and 8 days after the third immunization (Figure 2A). Serological responses were compared by first measuring the total Env-specific IgG binding titers after the immunizations. Following the second immunization, there was a clear difference in the total Env-specific IgG response between the two groups with higher responses detected in mice immunized with unfixed 16055 NFL TD CC Env trimers compared to those immunized with fixed trimers as shown by titration curves of the individual mice (Figure S1A in Supplementary Material) and as group means (Figure 2B). While this difference was detectable using both unfixed and GLA-fixed trimers as the antigenic target in the ELISA assay, it was more apparent when the unfixed protein was used for coating. After the third immunization, only a modest difference remained between the groups when the unfixed protein was used for coating the ELISA plates (Figure 2B), and an even smaller difference was observed when the fixed protein was used for coating (Figure 2C). Control mice injected with adjuvant alone showed no Env-specific binding.

### Env-Specific Antibody Subclass Responses in Mice Immunized with Unfixed or GLA-Fixed Trimers

**Figure 3:**
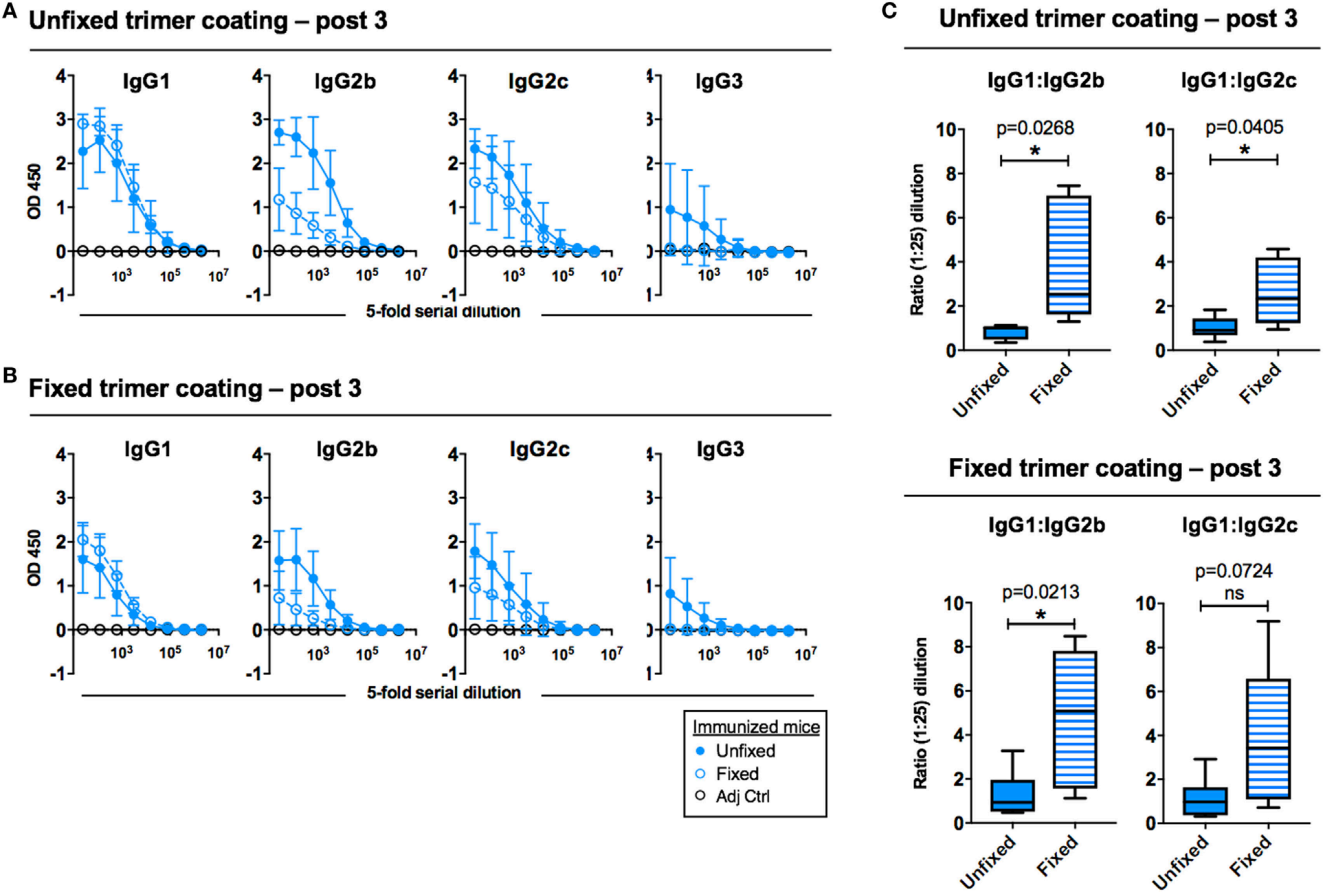
Env-specific antibody subclass responses after three immunizations measured by ELISA. **(A)** ELISA curves (group means) for Env-specific IgG1, IgG2b, IgG2c, and IgG3 using plates coated with unfixed 16055 native flexibly linked (NFL) trimer-derived (TD) CC Env trimers. **(B)** ELISA curves (group means) for Env-specific IgG1, IgG2b, IgG2c, and IgG3 using plates coated with fixed 16055 NFL TD CC Env trimers. **(C)** The ratio of IgG1:IgG2b and IgG1:IgG2c were compared for unfixed protein coating (top) and glutaraldehyde-fixed protein coating (bottom). * and ** indicate statistical significance (Student’s *t*-test) between mice immunized with unfixed and fixed 16055 NFL TD CC Env trimers. Fivefold serial dilution was used for all samples starting at a 1:25 dilution.

To investigate potential qualitative differences in the response elicited by the unfixed and GLA-fixed 16055 NFL TD CC Env trimers, we assessed the elicited serum IgG subclass response after three immunizations. We observed that mice immunized with fixed Env trimers generated a different subclass pattern compared to the mice immunized with unfixed trimers. Specifically, while the IgG1 responses were similar, mice immunized with fixed trimers displayed lower IgG2b, IgG2c, and IgG3 titers compared to mice immunized with the unfixed trimers, independently of whether unfixed protein (Figure 3A) or fixed protein (Figure 3B) was used as the binding target on the ELISA plate. This difference was not observed when sera collected after two immunizations were analyzed, likely because the IgG subclass responses were still very low at this time point (Figure S1B in Supplementary Material). We also compared the ratios between the subclasses at a serum dilution of 1:25 and observed differences between the groups, which were significant for both IgG1:IgG2b and IgG1:IgG2c ratios when unfixed protein was used for coating (Figure 3C, upper panel) and for the IgG1:IgG2b ratio when fixed protein was used for coating (Figure 3C, lower panel). These serological results suggested a Th2-shifted response following immunization with the GLA-fixed 16055 NFL TD CC Env trimers. Control mice injected with adjuvant alone showed no Env-specific IgG1, IgG2a, IgG2c, or IgG3.

To determine if this difference was detected if additional boosts were performed, we performed an independent immunization experiment where mice were injected five sequential times with the unfixed or GLA-fixed 16055 NFL TD CC Env trimers in AbISCO-100. This experiment yielded very similar results with increased IgG1:IgG2b and IgG1:IgG2c ratios in mice immunized with GLA-fixed trimers compared to mice immunized with unfixed trimers. This difference was significant for the IgG1:IgG2b ratio using both unfixed and fixed protein for coating (Figure S2 in Supplementary Material).

### T Cell Responses Elicited in Mice Immunized with Unfixed or GLA-Fixed Trimers

**Figure 4:**
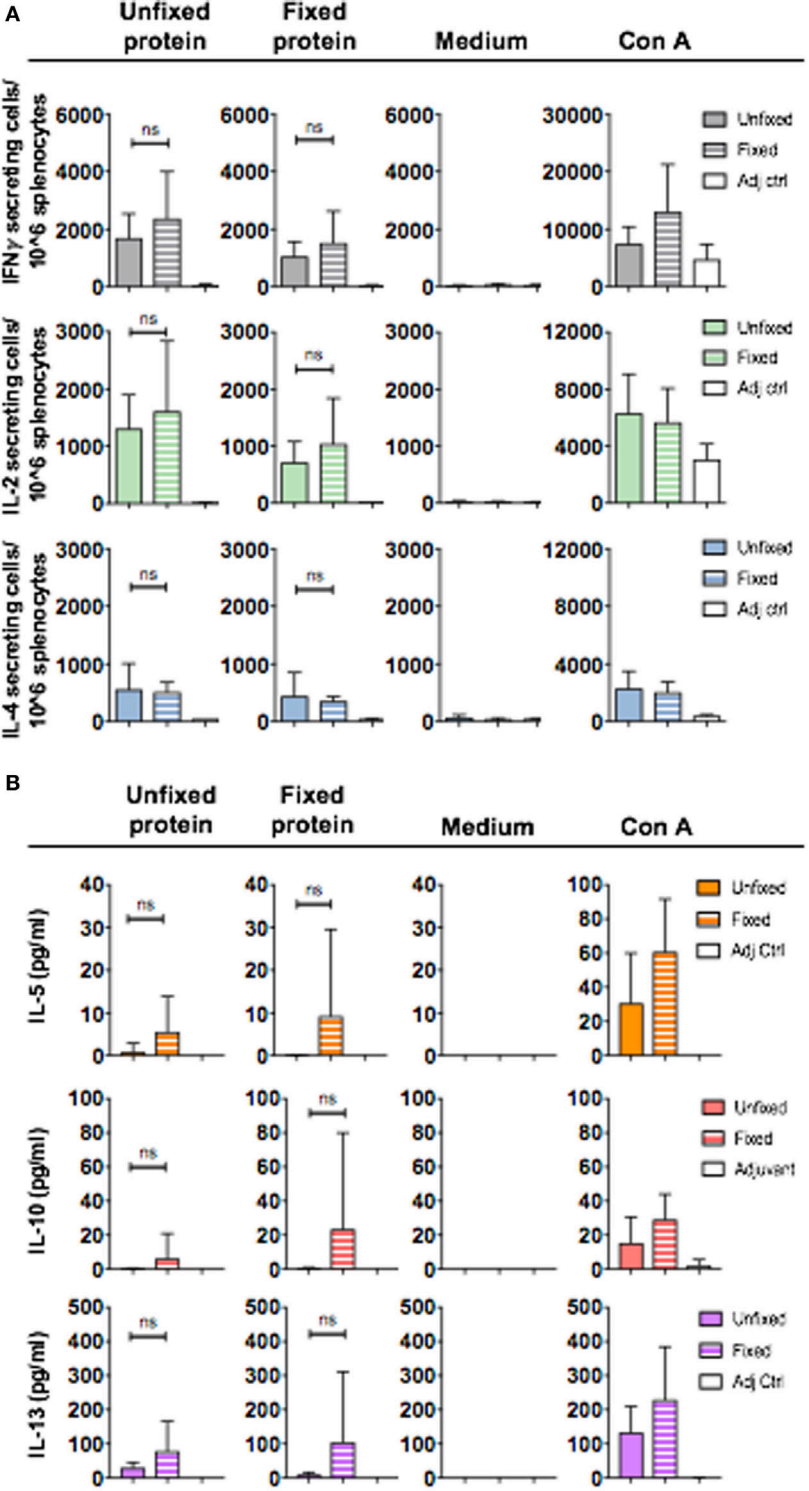
CD4+ T cell cytokine production after *in vitro* stimulation of splenocytes collected after three immunizations with unfixed or fixed 16055 native flexibly linked (NFL) trimer-derived (TD) CC Env trimers or adjuvant only. **(A)** IFNγ, IL-2, and IL-4 cytokine-producing cells (group means) were measured by ELISpot analysis after 20 h stimulation with unfixed or fixed Env trimers or with ConA (positive control) or medium (negative control). Each spot formed in the wells represents one cytokine-producing cell. The average number for cytokine-producing cells for each group was plotted for each stimulus. Statistical significance (Student’s *t*-test) between mice immunized with unfixed and fixed protein was tested. **(B)** Detection of IL-5, IL-10, and IL-13 in supernatants following 20 h *in vitro* stimulation with unfixed NFL TD CC trimers, glutaraldehyde-fixed NFL TD CC trimers, Con A (positive control), or medium (negative control) using a flow cytometry-based bead assay. The results were plotted as picograms per milliliter cytokine produced for each condition. The detection limit of cytokines was 0.273 pg/ml.

Having observed that unfixed and fixed 16055 NFL TD CC Env trimers induce qualitatively different IgG subclass responses, we next investigated whether the Env-specific T cells responses also differed between animals in each of the groups. We first used a cytokine ELISpot analysis of splenocytes harvested after the third immunization for this analysis. We evaluated if the response measured by our protein stimulation conditions (20 h at 37°C) resulted from CD4+ T cells by comparing cytokine production in total splenocytes to the CD4+ T cell-depleted splenocytes (Figure S3A in Supplementary Material). This experiment confirmed that both the IFNγ and IL-2 cytokine production measured in response to protein stimulation was CD4+ T cell-dependent as the CD4+ T cell-depleted samples did not secrete cytokine levels that exceeded those of the medium control (Figures S3B,C in Supplementary Material). We next applied this method to analyze spleens harvested from mice immunized three times with unfixed or GLA-fixed 16055 NFL TD CC trimers and detected no significant differences in the number of IFNγ, IL-2, and IL-4 producing T cells upon stimulation with NFL trimers (unfixed or fixed) (Figure 4A). We concluded that mice immunized with unfixed or GLA-fixed 16055 NFL TD CC Env trimers had similar numbers of cytokine-producing cells, indicating similar uptake and processing of the GLA-fixed and unfixed trimers by antigen-presenting cells for CD4+ T cell activation. To specifically investigate the presence of T cells producing Th2-associated cytokines, we employed a flow cytometry-based bead assay to detect low levels of cytokines potentially present in supernatants from *in vitro*-stimulated T cells from mice immunized three times with unfixed or GLA-fixed Env trimers. We detected varying levels of IL-5, IL-10, and IL-13 with several mice being under the limit of detection of the assay. However, the mice that did respond with detectable IL-5, IL-10, and IL-13 levels were almost exclusively found among the mice immunized with the GLA-fixed trimers, suggesting a potential connection to the antibody subclass response in these mice (Figure 4B).

## Discussion

Here, we performed a comparative study in mice to examine the magnitude and quality of the Env-specific immune responses induced by unfixed or GLA-fixed 16055 NFL TD CC trimers. We demonstrate that the GLA-fixed 16055 NFL TD CC trimers displayed increased thermostability *in vitro*, reduced exposure of non-neutralizing antibody epitopes *in vitro* and lower *in vivo* Env-specific IgG antibody responses after two immunizations. However, following three immunizations the difference in Env-specific IgG titers was modest and only detectable when unfixed trimers were used as the target antigen in the ELISA. The difference in magnitude of the response induced by the unfixed trimers compared to the fixed trimers may be because unfixed trimers are more prone to unfolding or dissociation *in vivo*, resulting in exposure of immunogenic but non-neutralizing protein determinants. Thus, a reduced response may be a desired outcome if it means that less antibody responses to irrelevant epitopes. Induction of antibodies against non-desired, non-neutralizing epitopes may register by ELISA when unfixed trimers are used for coating but less so when the fixed protein is used. Thus, the use of both unfixed and fixed trimers as both immunogens and as binding targets in the ELISA plate provides useful information to deduce trends in the elicited antibody specificities *in vivo*. Whether GLA fixation offers an advantage for the quality of neutralizing antibody responses induced by the 16055 NFL TD CC trimers was not addressed in the present study, as this was previously investigated in the guinea pig model (7), as well as in the rabbit model using other well-ordered Env trimer designs (9, 10). We have previously reported tier 1 neutralizing activity in immunized mice (12, 13). However, immunogens that readily elicits tier 2 neutralizing antibody titers in rabbits (9, 10, 14) fail to do so in mice (15, 16).

The most significant observation in this study was that the Env-specific IgG response measured in animals inoculated with GLA-fixed trimers displayed a more Th2-skewed subclass profile than the response elicited in mice inoculated with unfixed trimers. We demonstrated this outcome by measuring Env-specific IgG1, IgG2b, and IgG2c. In mice, IgG1 represents a Th2-skewed response, while IgG2b and IgG2c represent a Th1-skewed response. A previous study in Balb/c mice reported that Env delivered in the form of a DNA vaccine induced a more Th2-biased antibody subclass response profile than did a DNA-based influenza virus hemagglutinin-based vaccine, as detected by an increased IgG1:IgG2a ratio in mice immunized with Env (17). This suggested an intrinsic difference in the type of response induced by the two viral antigens. We previously examined antibody subclass profiles induced by purified HIV-1 Env trimers formulated in the AbISCO-100 adjuvant in a head-to-head comparison between Balb/c mice and C57BL/6 mice and found that balanced Th1/Th2 responses were induced in both strains, with potent Env-specific IgG1, IgG2a, and IgG2b responses detected in Balb/c mice and similarly potent IgG1, IgG2b, and IgG2c responses detected in C57BL/6 mice (13). In the current study, we detected potent IgG1, IgG2b, and IgG2c responses to the unfixed trimers but reduced IgG2b and IgG2c responses to the fixed trimers. This Th2 skewing of the antibody subclass responses was observed in all animals immunized with the GLA-fixed trimers, using either the unfixed or GLA-fixed Env trimers as the antigenic target coated on the ELISA plates and was observed in two independent experiments.

The unfixed and GLA-fixed trimers used here were formulated with the AbISCO-100 adjuvant (also called Matrix-M). We and others have previously shown that this adjuvant induces a balanced Th1/Th2 response also for other protein antigens (18–20). Our results show that the presence of GLA adducts on the trimers influenced the induced immune response in a manner that was not over-ridden by the presence of the adjuvant. We have previously shown that the response induced by protein antigens in AbISCO-100 can be shifted toward a more Th1-driven response by co-administration of a TLR9 agonist (18). Thus, co-stimulation of TLR9 may be one way to balance the Th2 skewing caused by the GLA fixation. Another strategy to direct the Env-specific response away from a Th2-biased profile is to prime with a viral vector expressing Env prior to protein boosting, which we previously showed induced a more Th1-biased response (21). While the assessment of Th1/Th2 skewing by measurements of IgG subclasses may not be readily translatable to other species, our finding may be worthy of further investigation in other models using additional assays of T helper function as different vaccine platforms are under evaluation and prioritization. While formaldehyde treatment of proteins was shown to limit antigen processing by constraining presentation to T cells in one study (22), we did not detect any measurable differences in the magnitude of cytokine-producing profiles of Env-specific CD4+ T cells in our study using unfixed or GLA-fixed protein for *in vitro* stimulation when IFNγ, IL-2, and IL-4 cytokine responses were measured by ELISPOT analysis. When a more sensitive flow cytometry-based bead assay was used to detect additional Th2 cytokines, we observed that the highest IL-5, IL-10, and IL-13 responders were found in the group of mice immunized with the GLA-fixed trimers. This result was non-significant since a majority of the mice were below the level of detection. Nevertheless, it indicated a trend toward an increased Th2 response after immunization with fixed trimers, which may be related to the skewed antibody subclass response.

Chemical fixation of viruses and antigens is used in some commercial vaccines, for example, to inactivate infection by replication-competent whole virus particles. This was successfully done for the polio vaccine but it was less successful for a candidate respiratory syncytial virus (RSV) vaccine (23). In the case of the clinically tested RSV vaccine, formaldehyde was used for fixation. This vaccine worsened clinical symptoms in children exposed to natural RSV infection, triggering its removal from commercial development (24). The negative outcome was associated with a shift in the responses from a Th1 to a Th2 profile, as well as lower levels of neutralizing antibodies (24–26), effects that were suggested to be related to the carbonyl groups on the vaccine antigens (27). These studies, as well as those presented here, indicate the need for an improved understanding of how modifications to protein-based vaccines influence the induced response at multiple levels. For example, protein adducts such as aldehyde groups may interact with scavenger receptors such as CD36 (28), which are expressed by both B cells and professional antigen-presenting cells. Whether such potential effects influence antigen-specific immune responses to HIV-1 Env or other viral proteins is not known, but may be elucidated by future investigations.

## Ethics Statement

All animal experiments were performed under approved conditions and standard guidelines prior to the experimental start according to the regulations of the Committee for Animal Ethics (Stockholm, Sweden). The ethical permit number is N4/16.

## Author Contributions

MS: planning and performing *in vivo* experiments, data collection, data analysis, making figures, and writing manuscript. MA: performing *in vivo* experiments, data collection, and reviewing manuscript. JS: planning, performing, data collection and data analysis in T cell in vitro experiments. LY: preparing recombinant proteins for EM and performing EM analysis. YF, KT, RW, and JG: recombinant protein production and characterization, data analysis, making figures, and reviewing manuscript. RTW: planning the study and supervision and reviewing of manuscript. GKH: study planning, supervision, and writing manuscript.

## Conflict of Interest Statement

The authors declare that the research was conducted in the absence of any commercial or financial relationships that could be construed as a potential conflict of interest.

## Supplementary Material

Figure S1 Total Env-specific IgG and subclass-specific responses measured by ELISA. **(A)** ELISA curves for Env-specific IgG for individual mice (post-2 and post-3) using unfixed 16055 native flexibly linked (NFL) trimer-derived (TD) CC Env trimers (left) or fixed 16055 NFL TD CC Env trimers (right) as the target antigen. **(B)** ELISA curves (group means) for Env-specific IgG1, IgG2b, IgG2c, and IgG3 from post-2 sera using fixed 16055 NFL TD CC Env trimers as the target antigen. Fivefold serial dilution was used for all samples starting at a 1:25 dilution.

Figure S2 Env-specific antibody subclass responses after five immunizations measured by ELISA. **(A)** ELISA curves (group means) for Env-specific IgG1, IgG2b, and IgG2c using unfixed 16055 native flexibly linked (NFL) trimer-derived (TD) CC Env trimers as target antigen. **(B)** ELISA curves (group means) for Env-specific IgG1, IgG2b, and IgG2c using fixed 16055 NFL TD CC Env trimers as target antigen. **(C)** Ratios of IgG1:IgG2b and IgG1:IgG2c calculated using either unfixed and glutaraldehyde-fixed protein coated on the ELISA plates. The serum samples were added to the plates in fivefold serial dilutions starting at 1:50. * and ** indicate statistical significance (Student’s *t*-test) between mice immunized with unfixed and fixed 16055 NFL TD CC Env trimers.

Figure S3 CD4+ T cell depletion of splenocytes. **(A)** Assessment of the purity after CD4+ T cell depletion. The FACS plots show the changes in CD4, CD8, and B220 cell populations before and after CD4 depletion. IFNγ **(B)** and IL-2 **(C)** cytokine-producing cells were measured by ELISpot 20 h after stimulation with unfixed 16055 NFL TD CC trimers or with Con A (positive control) or medium (negative control) of splenocytes with or without CD4+ T cell depletion.
