# Supplementary figures and images for: Glutaraldehyde Cross-linking of HIV-1 Env Trimers Skews the Antibody Subclass Response in Mice
Source: Front Immunol. 2017 Nov 27;8:1654. doi: 10.3389/fimmu.2017.01654 (PMC5711779; doi:10.3389/fimmu.2017.01654)

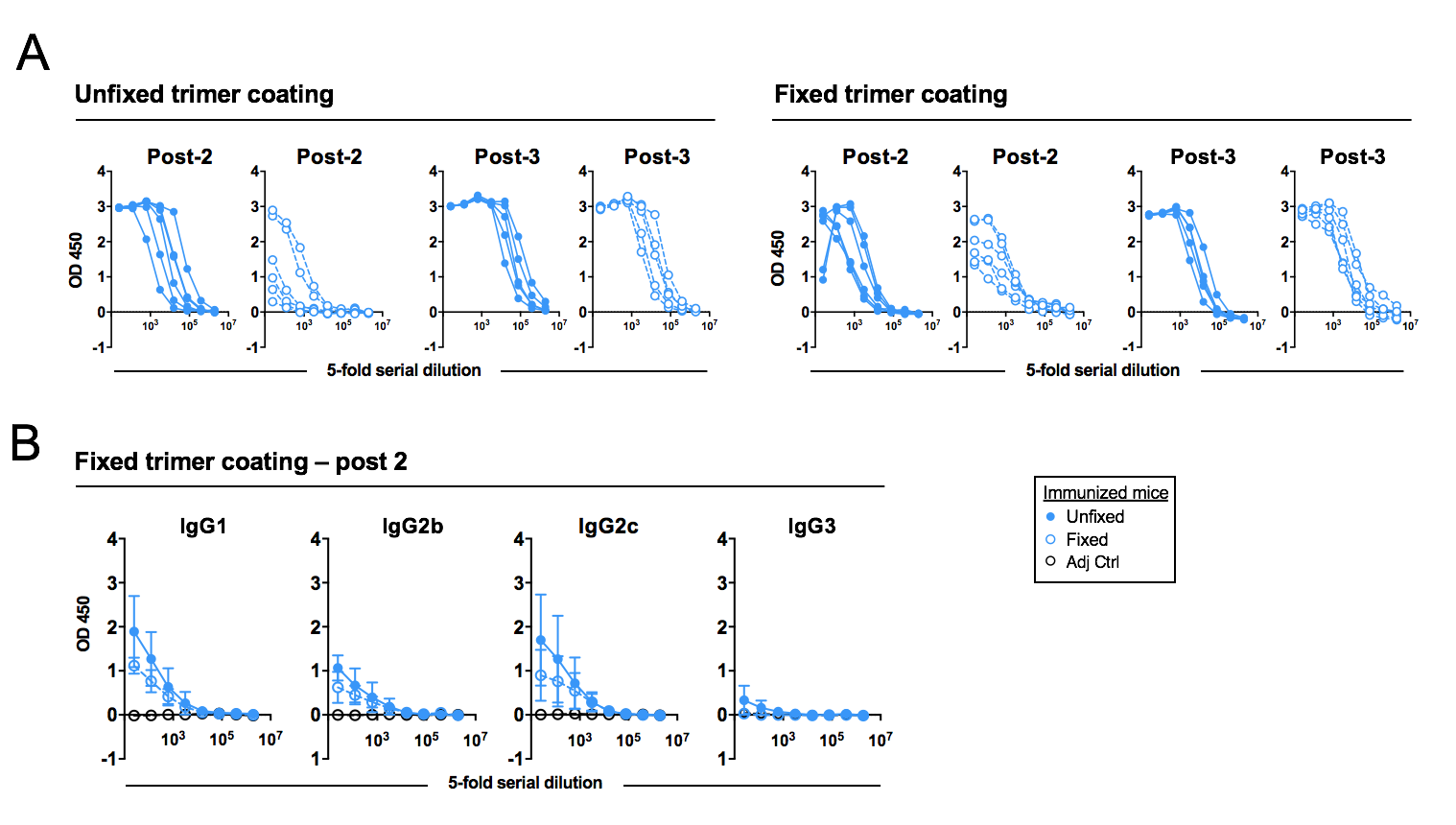

Supplement: Figure S1 — Total Env-specific IgG and subclass-specific responses measured by ELISA. (A) ELISA curves for Env-specific IgG for individual mice (post-2 and post-3) using unfixed 16055 native flexibly linked (NFL) trimer-derived (TD) CC Env trimers (left) or fixed 16055 NFL TD CC Env trimers (right) as the target antigen. (B) ELISA curves (group means) for Env-specific IgG1, IgG2b, IgG2c, and IgG3 from post-2 sera using fixed 16055 NFL TD CC Env trimers as the target antigen. Fivefold serial dilution was used for all samples starting at a 1:25 dilution. [file Image_1.TIFF]

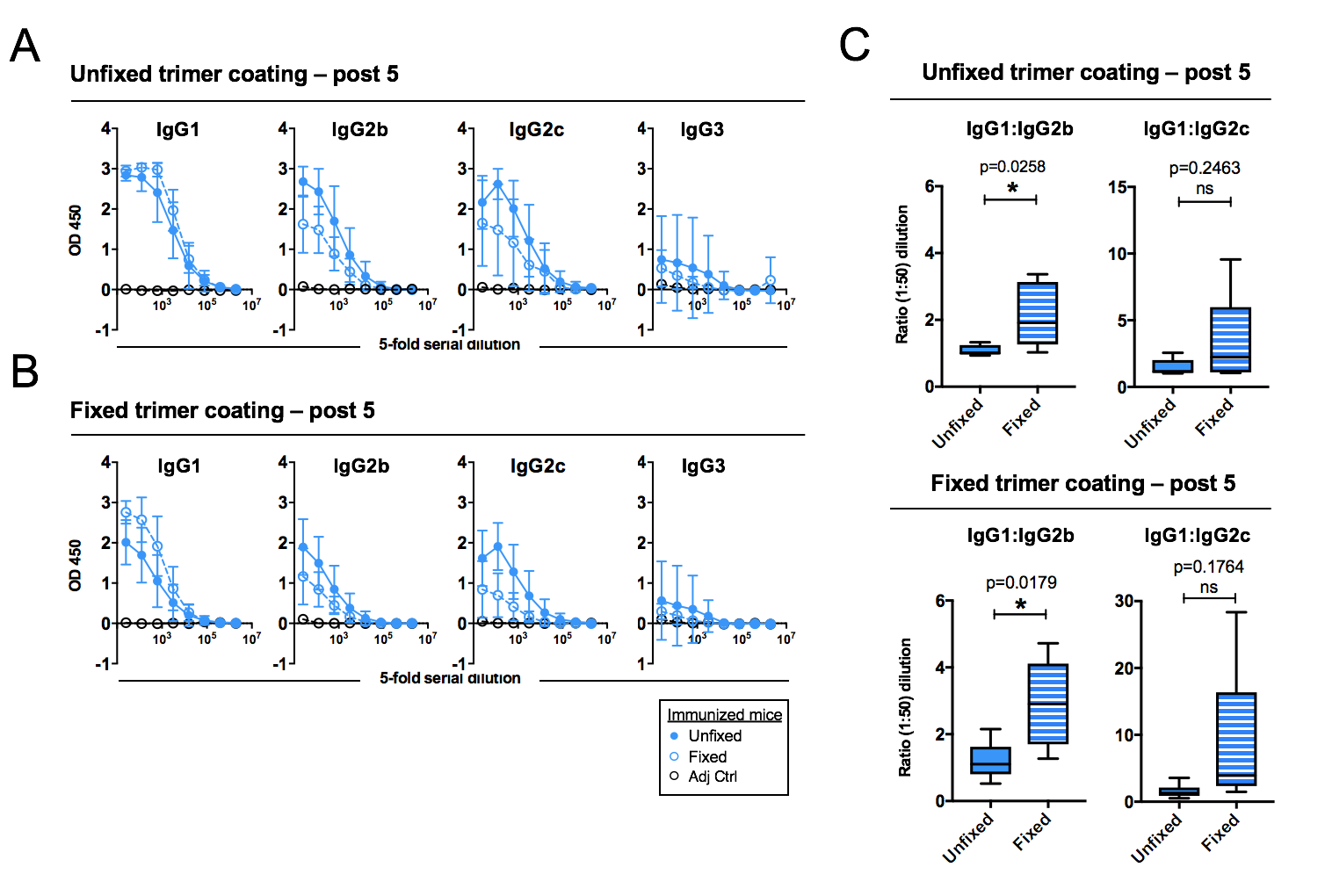

Supplement: Figure S2 — Env-specific antibody subclass responses after five immunizations measured by ELISA. (A) ELISA curves (group means) for Env-specific IgG1, IgG2b, and IgG2c using unfixed 16055 native flexibly linked (NFL) trimer-derived (TD) CC Env trimers as target antigen. (B) ELISA curves (group means) for Env-specific IgG1, IgG2b, and IgG2c using fixed 16055 NFL TD CC Env trimers as target antigen. (C) Ratios of IgG1:IgG2b and IgG1:IgG2c calculated using either unfixed and glutaraldehyde-fixed protein coated on the ELISA plates. The serum samples were added to the plates in fivefold serial dilutions starting at 1:50. * and ** indicate statistical significance (Student’s t-test) between mice immunized with unfixed and fixed 16055 NFL TD CC Env trimers. [file Image_2.TIFF]

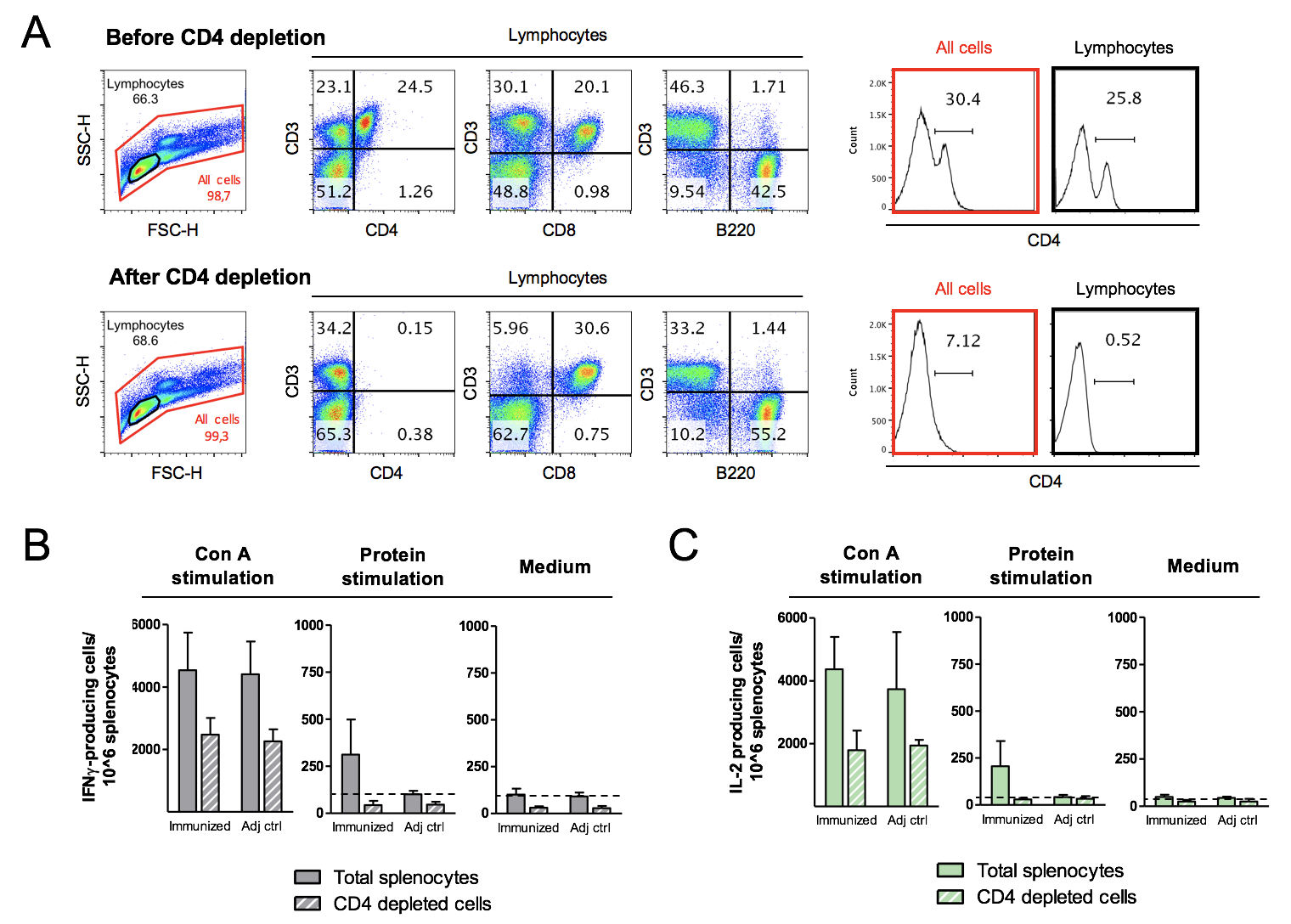

Supplement: Figure S3 — CD4+ T cell depletion of splenocytes. (A) Assessment of the purity after CD4+ T cell depletion. The FACS plots show the changes in CD4, CD8, and B220 cell populations before and after CD4 depletion. IFNγ (B) and IL-2 (C) cytokine-producing cells were measured by ELISpot 20 h after stimulation with unfixed 16055 NFL TD CC trimers or with Con A (positive control) or medium (negative control) of splenocytes with or without CD4+ T cell depletion. [file Image_3.TIFF]
